# Serum miRNA-101 expression signature as non-invasive diagnostic biomarker for Hepatitis C virus-associated hepatocellular carcinoma in Egyptian patients

**DOI:** 10.1038/s41598-024-81207-2

**Published:** 2025-01-03

**Authors:** Mostafa A. Sharafeldin, Reda A. Suef, Adel A. Mousa, Dina H. Ziada, Mohamed M. S. Farag

**Affiliations:** 1 Botany and Microbiology Department, Faculty of Science, Al-Azhar University, Cairo, 11884 Egypt; 2 Department of Tropical Medicine and Infectious Diseases, Faculty of Medicine, Tanta University, Tanta, Egypt; 3 Biomedical Research Department, Armed Forces College of Medicine (AFCM), Cairo, Egypt; 4 The Regional Centre for Mycology and Biotechnology, Al-Azhar University, Cairo, Egypt

**Keywords:** Hepatitis C virus (HCV), Hepatocellular carcinoma (HCC), miRNA-101 expression, AFP, Noninvasive Diagnostic Biomarkers, Therapeutic Targets, Cancer, Microbiology, Molecular biology, Biomarkers, Medical research, Molecular medicine, Oncology

## Abstract

Hepatocellular carcinoma (HCC) is a leading cause of cancer mortality globally due to HCC late diagnosis and limited treatment options. MiRNAs (miRNAs) emerged as potential biomarkers for various diseases, including HCC. However, the value of miRNA-101 as a serum biomarker for HCV-induced HCC has not been fully investigated. Our study aims to investigate the miRNA-101 differential expression in Egyptian HCV-induced HCC patients’ serum versus HCV liver cirrhosis (LC) as prospective diagnostic biomarkers compared to alpha-fetoprotein (AFP). Blood samples were collected for clinical chemistry profile, liver function, and serum AFP investigations. The serum miR-101 expression levels were evaluated using real-time quantitative PCR (RT-qPCR) in 100 Egyptian subjects: 40 HCV-induced HCC, 40 HCV-induced cirrhosis, and 20 healthy controls. HCC patients showed significantly higher TB, DB, and AFP levels than those cirrhosis and control groups, whereas ALB and Total Protein exhibited significantly reduced levels. AFP sensitivity and specificity in differentiating HCC reported 60 and 67%, respectively, at the cut-off values of 7ng/dl. miR-101 shows fold change upregulation in HCC patients (P < 0.0001) compared to LC and control groups. ROC curve demonstrated miR-101 (AUC) of 0.9556, sensitivity 92.5%, and specificity 97.5%, highlighting the miR-101 diagnostic potential as a biomarker for HCC detection. Elevated miR-101 levels in HCC are significantly correlated with a higher number and larger size of focal lesions, advanced BCLC staging, and Child–Pugh score. These findings highlight the utility of miR-101 as a predictive and diagnostic non-invasive biomarker for HCV-related HCC from cirrhotic populations. More research is warranted to validate the clinical validity of miR-101 and explore underlying mechanisms in HCV-HCC progression.

## Introduction

Hepatocellular carcinoma (HCC) is the fifth most common cancer globally^1^, with hepatitis C virus (HCV), as the main risk factor for contributing to around 25% of HCC cases. In Egypt, the prevalence of HCV infection (annual incidence rate of 170,000 new infections each year) has significantly increased the incidence of HCC over the past decade^2^.

Early detection of HCC is crucial in high-risk patients. However, current diagnostic methods rely on costly imaging techniques using triphasic computed tomography (CT) or dynamic magnetic resonance imaging (MRI)^3^, with variable accuracy depending on tumor size and vascularity^4^. Despite the commonly used Alpha-fetoprotein (AFP) as a serum tumor marker, for diagnosis and follow-up of HCV-associated HCC in high-risk individuals^5^, the predictive performance reported increased AFP levels in 60–70% of HCC patients with cirrhosis^6^. Furthermore, approximately 30—40% of early-stage HCC cases with small tumors reported normal serum AFP levels^7^. Regarding the diagnostic performance, AFP demonstrates inadequate sensitivities (41 to 65%), and specificities (80 to 94%) at a cut-off of 20 ng/mL for HCC at any stage in cirrhotic patients^8^. To develop efficient non-invasive biomarkers for early HCC diagnosis, other targets of circulating blood protein biomarkers have been examined^9^. However, limited candidates have shown sufficient accuracy to be considered optional by international organizations^10^.

In recent years, there has been a growing focus on the use of genetic biomarkers such as miRNAs due to their potential to be used as an effective diagnostic tool in the field of molecular oncology^11^. miRNAs, a type of small noncoding RNAs ranging from 19 to 25 nucleotides in length^12^, play a regulatory post-transcriptional role in gene expression by inhibiting target genes at the mRNA level. This inhibition can lead to mRNA degradation or blockade of protein translation^13^. Furthermore, miRNAs have been found to regulate different physiological processes in mammals such as the cell cycle, apoptosis, metabolism, proliferation, and differentiation^14^. The modified miRNA expression is associated with various pathogenic events, including cancer onset, tumor development, apoptosis escape, and migration/invasion enhancement. Certain miRNAs act as oncogenes while others function as tumor suppressors^15^. In HCC, specific miRNAs are frequently downregulated or overexpressed^16^.

In the exploration of miRNAs as potential biomarkers for the early detection and diagnosis of HCV-associated HCC, a collective of 26 prospective candidates were recognized, either individually^17^, or in combination, according to bioinformatics analysis^18^. However, further research is required to establish the diagnostic validity of these specific miRNAs.

miR-101 is involved in various biological processes and its dysregulation has been linked to several forms of cancer^19,20^. A study reported an association between miR-101–1 polymorphisms, their upregulated expressions, and early prediction of HCC^21^. miR-101 has also been shown to be altered in patients with HBV-associated HCC in Egypt by exhibiting a down-regulated expression in HCC tissues but an up-regulated in serum, indicating a possible release from tumor cells into the bloodstream. Furthermore, serum miR-101 levels can discriminate HCC patients from healthy individuals or chronic HBV patients, suggesting its potential use as a biomarker for diagnosing HCC^22,23^. However, the focus of previous research is primarily on HBV-associated HCC, leaving a gap regarding the applicability of miR-101 to diagnosing HCV-associated HCC in Egypt. Additionally, these studies have not thoroughly evaluated the diagnostic accuracy of miR-101 compared to established tools such as AFP. Consequently, our study aims to address this knowledge gap by exploring miR-101’s diagnostic performance in chronic HCV-associated HCC in the Egyptian population, and its comparison with AFP, with the aim of improving the accuracy in discriminating cases of HCC.

## Materials and methods

### Study population

This retrospective case–control study aimed to investigate the diagnostic effect of miR-101 for diagnosed HCC HCV-induced HCC in comparison to AFP. In our study, 100 enrolled subjects, including 40 patients (23 male and 17 females, with varying stages of HCV-related HCC: 17 patients at early stage (**A**), 9 patients at intermediate stage (**B**), and 14 patients at advanced stage (**C**); 40 patients (26 male and 14 female) with HCV-related liver cirrhosis, and 20 (11 male and 9 female) HCV-free healthy subjects as a control group (HC). All participants were selected from inpatients and outpatient clinics from Tanta University Hospital, Tanta City, Egypt during the period from July 2019 to July 2022.

Comprehensive evaluations were conducted for all individuals, involving physical examination focused on identifying signs of decompensation such as ascites and jaundice. The historical assessment included factors such as family history, smoking, right hypochondrial pain, past occurrences of hepatic encephalopathy, hematemesis, and comorbidities such as diabetes mellitus. Radiological examinations and laboratory assessments including complete liver function tests (albumin, total bilirubin), direct bilirubin, ALT, and AST levels) as well as HBV and HCV serology were also performed in conjunction with HCV RT-PCR. The diagnosis of HCC is confirmed through a systematic approach. Initial screening involved measuring AFP levels and performing abdominal ultrasound. Suspected cases with hepatic focal lesions and / or elevated serum AFP levels, undergo additional dynamic imaging using triphasic computed tomography (CT) or dynamic magnetic resonance imaging (MRI). The key characteristics of typical HCC include the existence of nodules with enhanced arterial phase lesions, followed by early washout in the delayed phase. For cases presenting atypical features, a liver biopsy was undertaken to establish a definitive diagnosis. The Child–Pugh classification score was used to assess liver cirrhosis staging.^24,25^. The HCV-associated HCC group has been classified into five stages based on the Barcelona Clinic Liver Cancer (BCLC) staging system. BCLC strategy for prognosis prediction and treatment recommendation depends on the number, size, size of the tumor, vascular invasion preserved liver function, and performance state. Exclusion criteria included the presence of other HCV-induced liver diseases (HBV or alcohol), history of previous liver transplantation or malignancies other than HCC, prior treatment for HCC, or antiviral therapy for HCV within the last six months. Controls were carefully chosen based on similar criteria, except for lacking an HCC diagnosis. Written informed consent is obtained from participants prior to the commencement of the study. All laboratory tests were performed at the Central Laboratory, Tanta University Hospital. The study followed the criteria of the Helsinki Declaration and was approved by Tanta University Hospital’s ethical committee *(Approval Code: 36265MD195/2/24).*

### Laboratory assessment

Blood samples (5 mL blood/subject) were collected from patients at Tanta University Hospital using standard vein puncture techniques under sterilized conditions. Blood serum was obtained by centrifuging 3 ml at 3000 rpm for 15 min. The supernatant sera are collected and stored at -80 °C for complete blood picture, Laboratory chemistry, and liver function analysis including the serum AFP level, serum alanine transaminase (ALT), aspartate transaminase (AST), Albumin (IU/L), total and direct bilirubin, total protein, using commercially available assays. Also, two milliliters of blood were drawn into tubes containing EDTA for performing HCV RT-PCR, extracting RNA, and estimating the expression of the miRNA101 gene.

### Measurements of serum AFP level

Sera from HCC patients were used for the estimation of the serum level of AFP by ELISA, using the Bios Kit, USA as follows, the coated wells were filled with zero buffer, incubated, rinsed, and dried. The enzyme conjugate reagent was then added, incubated, and washed. The TMB substrate was added, incubated, and a stop solution was applied. The absorbance was measured at 450 nm using a microplate reader.

### Evaluation of miR-101 gene expression

miR-101 levels in blood were determined following the procedures outlined in the Direct-Zol™ RNA Miniprep Kit (ZYMO RESEARSH, USA). The sequential processes included three main steps: Serum preparation, miRNA101 extraction, cDNA synthesis, and RT-PCR.

#### Serum preparation and miR-101 extraction

In a vacutainer tube, two milliliters of blood were collected and centrifuged at 3000 rpm for 15 min at room temperature. The resulting supernatant was then poured into Eppendorf tubes. To remove cell debris, the samples were subjected to a second centrifugation at 15, 000 rpm for 15 min, and the resulting supernatants were stored for RNA extraction at -80°C.RNA was extracted from 50 μl of plasma utilizing a Direct-Zol™ RNA Miniprep Kit (ZYMO RESEARCH, USA), following the enrichment protocol for small RNA purification under the manufacturer’s instructions. Briefly, an equal volume of 95–100% ethanol was added to the TRI reagent sample and thoroughly mixed. After that, the mixture was centrifuged in a collecting tube and loaded to a Zymospin IICR column. The flow-through was disposed of and the column was relocated to a new collection tube. Subsequently, the column was loaded with 400 μl of RNA wash buffer and centrifuged. 5 μl of DNase 1 in an RNase-free tube (6 U/μl) was combined with 75 μl of DNA digestion buffer. The mixture was added directly to the column matrix and incubated at 20–30°C for 15 min. Next, 400 μl of Directzol RNA prewash was added to the column, centrifuged, and the flow-through discarded. Subsequently, the column was filled with 700 μl of RNA wash buffer and centrifuged for a minute. Lastly, a careful transfer of the column into an RNase-free tube was performed. After adding 50 μl of DNase/RNase-free water directly to the column matrix and centrifuging, the RNA was eluted. The quality of the RNA was checked with a NanoDrop2000 device (Thermo Scientific, USA).

#### Reverse transcription (RT)

Reverse transcription (RT) was performed using 5 μg per reaction using the TOPscriptTM RT DryMIX (dT18/dN6 Plus) Kit (Enzynomix, USA). According to the manufacturer’s instructions, the final volume of 20 μl RT reactions was loaded onto the thermal cycler under the following conditions: 25 °C for 10 min, 50 °C for 60 min, and 95 °C for 5 min to inactivate the reaction. The optimal reaction temperature ranged from 42 °C to 60 °C. Adhering to the manufacturer’s directions, the thermal cycler was loaded with 20 μl of RT reactions (final volume) and programmed as follows: 25 °C for 10 min, 50 °C for 60 min, and 95 °C for 5 min for reaction deactivation. The range of 42 °C to 60 °C was found to be the optimal reaction temperature.

#### Quantitative real-time polymerase chain reaction (RT-qPCR)

The miR-101 expression levels in serum were evaluated using a Quant studio 6 flex machine (Applied Biosystems) and the TOP real TM qPCR 2X PreMIX kit (SYBR Green with low ROX) (Enzynomix, USA). To ensure accurate measurements, the manufacturer’s protocol was followed. In both the control and patient sera, glyceraldehyde 3 phosphate dehydrogenase (GAPDH) was used as an internal control. The RT-qPCR mixture was prepared by combining 1 μl of RT products, 10 μl of TOP real qPCR SYBR Green Master Mix (2X), 1 μl of template DNA, 1 μl of forward primer (5–10 pmol/μl), 1 μl of reverse primer (5–10 pmol/μl), and up to 20 μl of sterile water (RNase-free). The miR101 sequence, as well as the sequences for endogenous controls, were obtained from the miR Base database. The miR-101 sequence was as follows: forward (F), AGGTGACTTCTTGTCCACGC, reverse (R), ATGGGGTTTTTCCACTGCCT. The GAPDH sequence was as follows: (F), GCAACTAGGATGGTGTGGCT (R), TCCCATTCCCCAGCTCTCATA^26^. PCR amplification was performed under the following settings: Initially, a denaturation cycle was performed at 95 °C for 10–15 min. Subsequently, a second denaturation cycle was conducted at 95 °C for 10 s. The annealing cycle was performed at 60 °C for 15 s. The elongation cycle was performed at 72 °C for 15–30 s. Finally, 45 cycles were conducted. The cycle threshold (Ct) in RT-qPCR measures the number of cycles required for the fluorescence signal to reach the threshold. To determine the fold change in miRNA expression levels based on real-time PCR data, the *Livak formula*, also known as 2^-ΔΔC^T, was used to compare target miRNA Ct values between a test sample and a calibrator sample, using a reference gene or miRNA for normalization. In this calculation, healthy controls were used as calibrators. The Livak formula involves the equation “ΔΔCt = [Ct (target, test) − Ct (reference, test)] − [Ct (target, calibrator) − Ct (reference, calibrator)^27^. This equation allows for the evaluation of relative changes in gene expression between the test and calibrator samples. A value of 1 indicates no change, values less than 1 indicate downregulation and values greater than 1 indicate upregulation.

### Statistical analysis

To verify the significance of variations in miRNA-101 expression between the HCV-HCC and control groups, statistical analyses were performed. The analysis was performed with GraphPad Prism Software (2012 v6.01 Inc., California, USA). All experiments were performed independently and in triplicate. After analysis, the data was shown as the mean ± standard Error (SE). Descriptive statistics were used to summarize the numerical and qualitative data. To compare the three groups, a one-way ANOVA variance analysis was performed. Additionally, a chi-square test was conducted to compare the two variables. To assess the diagnostic performance of miRNA101 in HCC serum. Receiver operating characteristics (ROC) analysis was used to predict the most effective cut-off values and assess the diagnostic significance of serum miRNA 101 in HCC with sensitivity plotted on the Y axis and 1-specificity on the X axis. Furthermore, the correlation between miRNA-101 expression and related HCC clinicopathological features was also investigated using Person’s correlation analysis.

## Results

### Demographic and laboratory profiles of the studied groups

Patient demographic characteristics of the three distinct groups are summarized in Table 1. For statistical analysis, there were statistically significant differences between the studied groups concerning age and gender. Males were predominant in the HCC, cirrhosis, and healthy control group. The mean age of HCV-associated HCC (60.90 ± 6.10). HCV-associated liver cirrhosis means age (60.03 years ± 8.41). Healthy controls were, on average, younger than patients with HCC and cirrhosis. The historical characteristics of the HCC analyzed group are displayed in Table 2.

**Table 1:** Demographic characteristics of different subjects under study.

*P* value (Age)	Cirrhotic	HCC	(HC)	Variables (mean ± SD)
	40	40	20	Number
< 0.0001	65 / 35	57.5 / 42.5	55 / 45	Gender, M/F (%)
(***)	60.03 ± 8.41	60.90 ± 6.10	30.00 ± 4.84	Age (years)
	39—78	51—72	21—38	Range

The ages are given as the means ± standard deviation.

*HC* healthy controls, *HCC* hepatocellular carcinoma, *M* Male, *F* Female.

**Table 2:** The historical characteristics of the analyzed group (n = 40).

	HCC patients (n) (%)		HCV-Cirrhosis (n) (%)	
FH of HCC	18	45%	6	15%
DM	18	45%	11	27.5%
Smoking	27	67.5%	17	42.5%
Coffee (3 Cups/day)	9	22.5%	21	52.5%
Hematemesis	16	40%	11	27.5%
Rt. hypochondrial pain	31	77.5%	9	22.5%
Jaundice	26	65%	22	55%

*FH* family history, *DM* diabetes mellitus.

Regarding the biochemical findings *analysis* for the studied groups, as presented in Table 3, the ALT and AST results indicate elevated levels of these enzymes which indicate disruption of liver cells. The statistical analysis shows no significant difference between HCV-associated HCC and cirrhotic compared to the control group. Patients with HCC and cirrhosis showed significantly lower albumin levels (P < 0.0001) compared to the control group with a substantial variation as lower in patients with HCC than the cirrhotic group when compared to control group. Total bilirubin levels in the investigated HCC and cirrhotic groups were high, with a significant difference across the groups (P < 0.0001), The difference between HCC and the other two groups was especially noticeable since HCC had the highest mean Total and direct bilirubin levels (3.5 ± 0.54; 1.945 ± 0.36) respectively. The control group had the highest mean level of Total Protein, followed by the cirrhotic group and finally the HCC group. Total protein levels vary significantly amongst the groups studied (*P* < 0.0001).

**Table 3:** The laboratory biochemical parameters among participant groups.

Clinical Chemistry	Denotation	HC	HCC	Cirrhotic	*P.* Value
ALT: (U/L)	Mean	20.1	37.125	36.825	0.2055 (ns)
	Min–max	11–31	9–225	9–157	
	SD	5.036	49.68	32.31	
AST: (U/L)	Mean	30.55	61.18	80.40	0.0612 (ns)
	Min–max	19—44	13—452	15—309	
	SD	6.134	102.1	62.88	
ALB (gm/dl)	Mean	4.505	2.2275	3.3125	** < 0.0001** ***
	Min–max	3.8–5.1	1–2.9	1.8–4.2	
	SD	0.41	0.40	0.45	
T. BiL (mg/dl)	Mean	0.84	3.5175	1.47	** < 0.0001**
	Min–max	0.6–1	2.5–5.1	0.8–6.7	
	SD	0.13	0.54	0.92	
D.BiL (mg/dl)	Mean	0.165	1.945	0.5025	** < 0.0001**
	Min–max	0.1–0.3	1.4–2.8	0.1–3.2	
	SD	0.06	0.36	0.49	
Total Protein (g/dl)	Mean	6.95	5.415	6.135	** < 0.0001**
	Min–max	6.4–7.4	3.1–6.6	5.1–6.8	
	SD	0.31	0.57	0.38	
	Mean	3.40	88.99	34.08	
AFP (ng/L)	Min–max	(1.0—6.7)	(1.8—873.1) ^**a, b**^	(1.0—177.1) ^**a**^	**0.0259**
	SD SE	1.74 0.39	186.9 29.55	50.40 7.97	*

The unpaired t-test: Groups marked by initial (a) are significantly different from control group. Groups marked by initial (b) are significantly different from HCV- cirrhotic group.

*ALT* alanine aminotransferase, *AST* aspartate aminotransferase, *TB* total bilirubin, *DB* direct bilirubin, *ALB* albumin, *ns* non-significant, SE Std. Error of Mean, *SD* Standard deviation, one way ANOVA test used for significance analysis between the groups, For the statistical variances between the groups under examination, Bold values are emphasized.

P. Value significant levels; > 0.05 (non-significant), < 0.05 (low (*), < 0.01 Moderate (**), < 0.001 High (***).

### AFP serum level detection in study groups

**Fig. 1:**
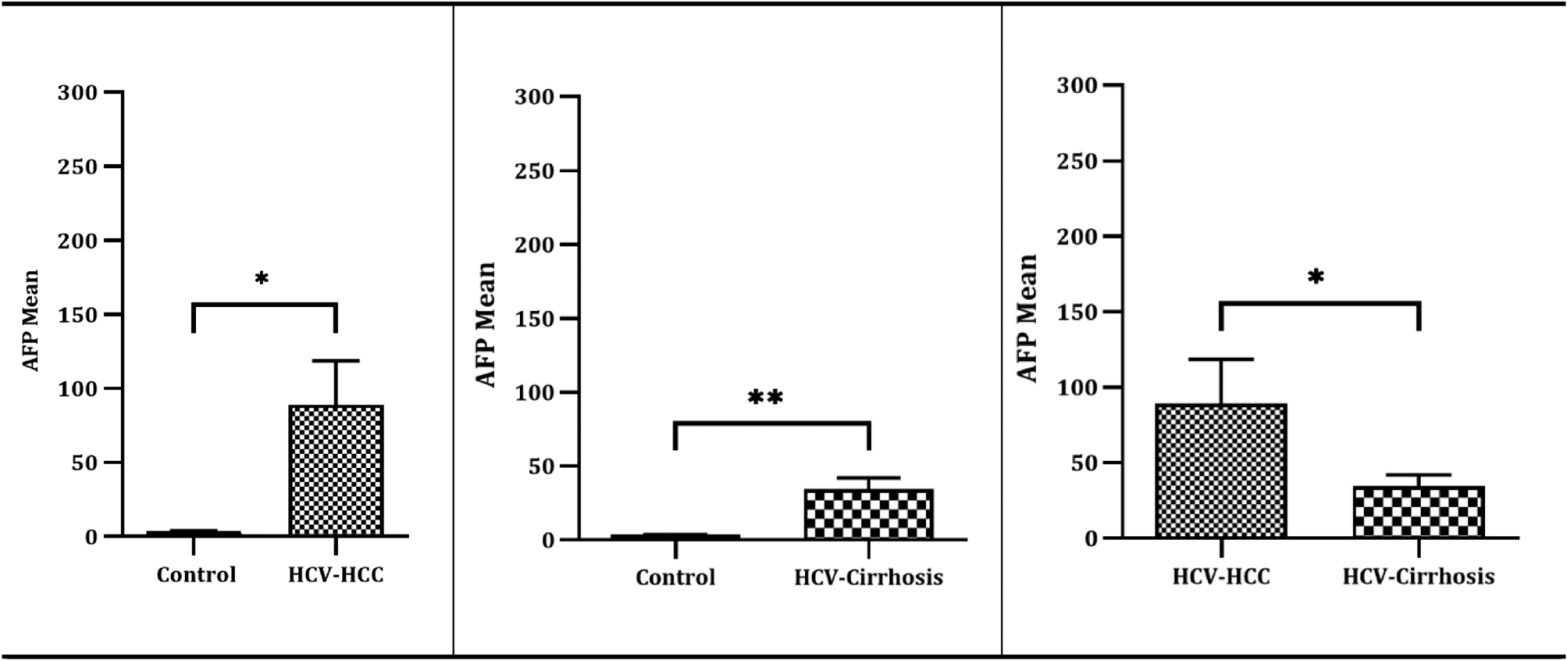
AFP Marker statistical analysis across study groups and control. Three separate replicates’ mean ± SE are displayed for each set of data. *P. Value* significant levels < 0.05 (low (*), < 0.01 Moderate (**), The unpaired t-test used for significance analysis between the groups.

Referring to the description of the average serum AFP biomarker levels in Table 3, analysis using ANOVA revealed statistically significant differences in the mean serum level of AFP among the three groups: the HCC group, the cirrhotic group, concerning the control group (*P* = 0.0230) with the highest mean level in HCC (88.99 ± 186.9). The unpaired t-test reported elevated AFP mean levels in HCV-associated HCC with a statistically significant difference in the mean serum AFP level when compared to both the HC group (*P* = 0.0230) and individuals in the cirrhotic (*P* = 0.0384). The cirrhotic groups reported elevated AFP mean levels with a statistically significant difference when compared to HC group (*P* = 0.0044) (Table 3, Fig. 1).

### Tumor characteristics within the HCC group

**Fig. 2:**
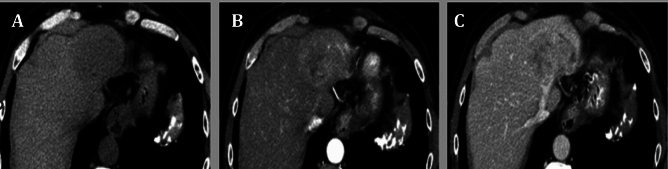
Triphasic CT images showed Large left lobe hepatic focal lesion (**A**) showed arterial enhancement (**B**) with rapid washout in delayed phase (**C**).

Regarding tumor characteristics within the HCV-associated HCC group (detailed in Table 4), a substantial majority of the patients (60%) fell into grade B as per Child–Pugh classifications, while according to BCLC staging, there were 17 patients in stage A (42.5%), 9 in stage B (22.5%), and 14 in stage C (35%) (Table 4). Patients with suspected hepatic focal lesions and/or elevated serum AFP levels underwent dynamic imaging using triphasic computed tomography. The results of the triphasic CT revealed characteristic features of HCC, including the presence of a large left lobe hepatic focal lesion (Fig. 2A) that showed arterial enhancement (Fig. 2B), followed by rapid washout in the delayed phase (Fig. 2C).

**Table 4:** The Tumors Characteristic of HCC studied cases based on the Barcelona Clinic Liver Cancer system (n = 40).

Characteristics	HCC [n (%)]
Child–Pugh score	
Stage A	16 (40%)
Stage B	24 (60%)
Ascites	
No	30 (75%)
Yes	10 (25%)
Hepatic encephalopathy	10 (25%)
Total numbers of tumors	
Single	29 (72.5%)
2 tumors	7 (12.5%)
3 tumors	4 (10%)
Size of the tumour (cm)/ CT	
1–3	28 (70%)
> 3	12 (30%)
BCLC	
Stage A	17 (42.5%)
Stage B	9 (22.5%)
Stage C	14 (35%)
Location of the focal lesions	
Rt lobe	32 (58%)
Lt lobe	23(42%)
Platelets (10^4^/mm^3^)	10.5 (2.7–25.3)
Prothrombin time %	88 (62–100)

Quantitative data is displayed as median (minimum–maximum). Qualitative data is expressed as numerical values and percentages.

*CT* computed tomography, *BCLC* Barcelona Clinic Liver Cancer, *Rt* right, *Lt* lift

### MiR-101 serum level detection in the investigated groups

**Fig. 3:**
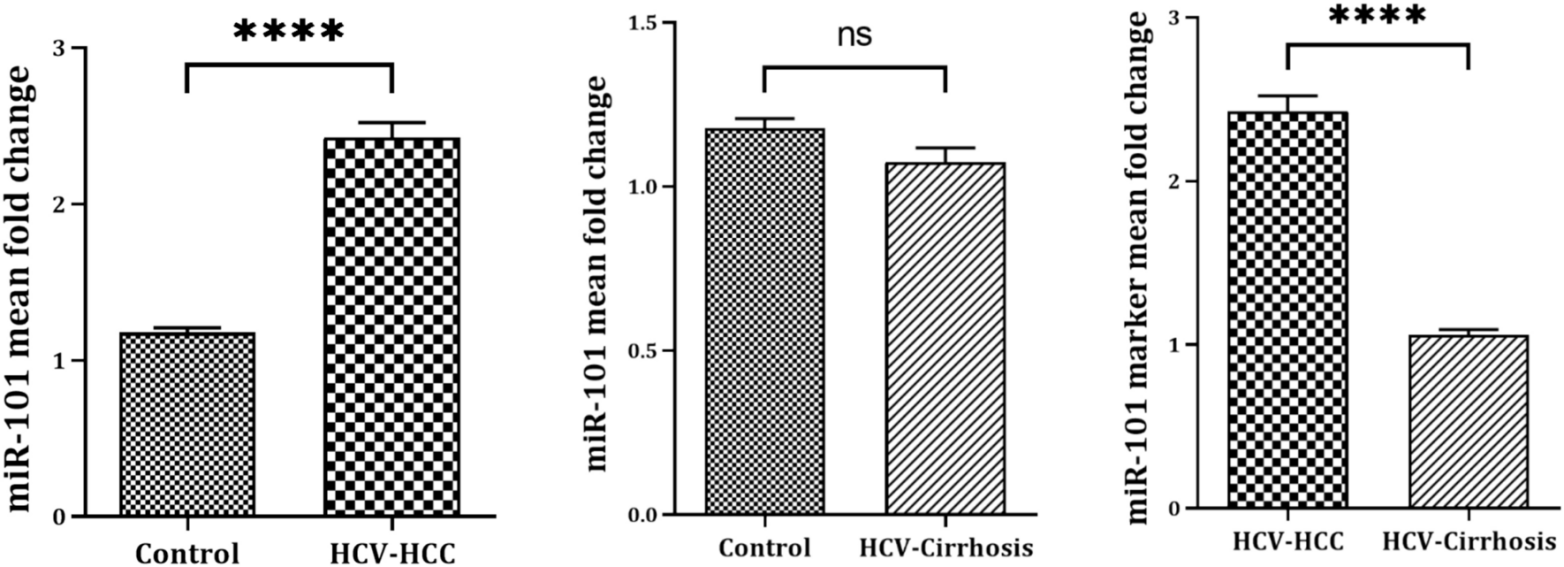
miR-101 marker mean fold change values for differentiating HCV- HCC from HCV-liver cirrhotic group and control. Three separate replicates’ mean ± SE are displayed for each set of data. P. Value significant levels > 0.05 (ns), < 0.0001 high (****), The unpaired t-test used for significance analysis between the groups.

The RT-qPCR fold change (FC) assessment for miR-101 expression in serum across HCV-associated HCC, HCV-liver cirrhosis, and control revealed a statistically significant difference (*P* < 0.0001). According to mean values FC, the serum level of miR-101in HCC patients revealed a highly statistically significant upregulated expression (*P* < 0.0001) of miR-101 compared to healthy controls (2.427 ± 0.597vs. 1.179 ± 0.13, representing a 2.4 -fold increase). On the other hand, comparing cirrhotic patients with the control group reported non-significant downregulated expression of miR-101 (1.062 ± 0.264 vs. 1.179 ± 0.13). Comparing the mean values of FC of miR-101 between the HCC group and the cirrhotic group revealed a significant difference in miR-101 levels, according to the Unpaired T-test (*P* < 0.0001) (Table 5, Fig. 3).

**Table 5:** Comparison of median values of fold change of studied miRNA -101 as marker for differentiating HCC group from cirrhotic groups

Denotation	miRNA-101 FC (2^-ΔC^T)			*P*. Value ANOVA	*P* value (unpaired t-test)		
	Control *(n* = 20)	HCC (*n* = 40)	Cirrhotic (*n* = 40)		HCC vs HC	Cirrhotic vs HC	HCC vs Cirrhotic
Mean ± SD	1.179 ± 0.13	2.427 ± 0.597	1.062 ± 0.264	< 0.0001***	** < 0.0001**	0.0527 (ns)	** < 0.0001**
Median	1.150	2.330	1.035				
Min–Max SE	1.0 – 1.34 0.02	1.03 – 3.31 0.09	0.84 – 2.01 0.04				

Fold Change represents the variations in levels of differentially expressed miRNA-101 in HCV-associated liver diseases between the studied groups and the control. SE Std. Error of Mean SD, standard deviation; miRNA, microRNA

### The diagnostic performance of serum AFP and miR-101 biomarkers

**Fig. 4:**
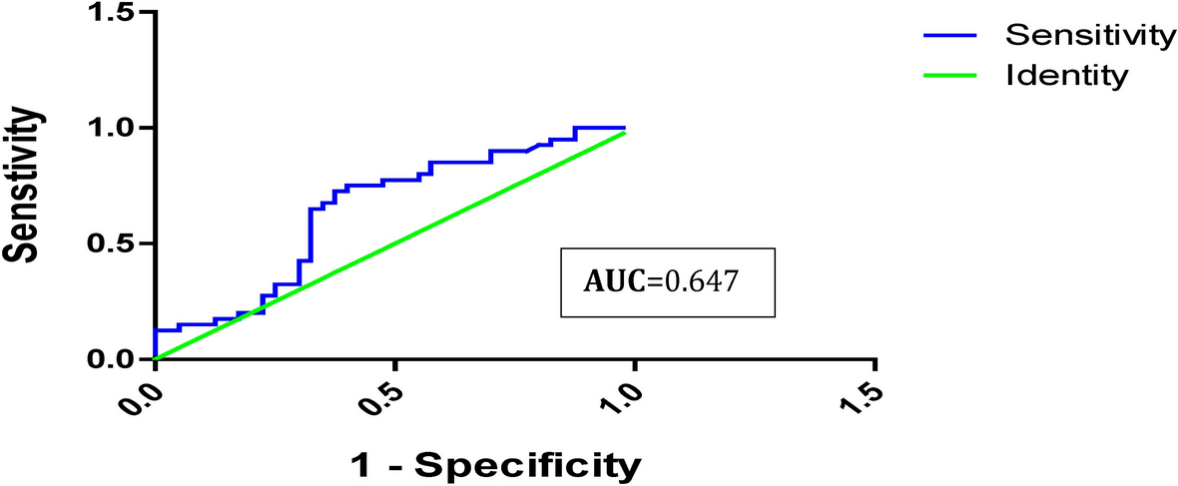
Serum AFP- ROC curve analysis for distinguishing HCV- HCC group from the HCV-liver cirrhotic and control groups.

**Fig. 5:**
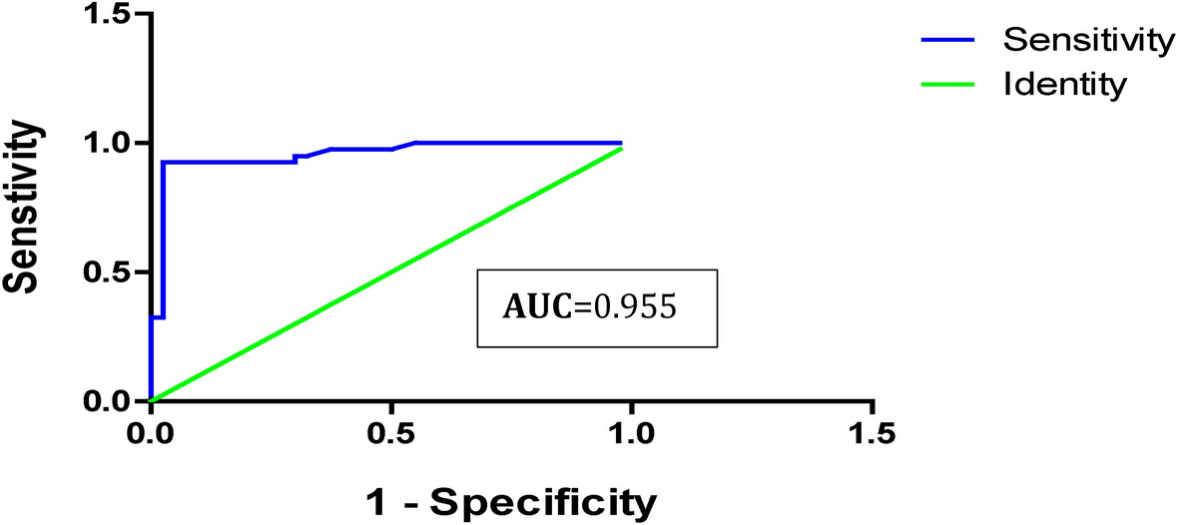
ROC curve analysis of miR-101 as an HCV- HCC tumor marker.

The ROC curves of serum AFP and miRNA-101 markers are provided in Table 6. AFP has a sensitivity of 60% and a specificity of 67% with a 7 ng/dl cut-off value, with a low validity ROC curve (AUC) = 0.6(95% CI: 0.524 — 0.770) of use in discriminating marker for HCC vs cirrhotic population (Table 6; Fig. 4). On the other hand, miRNA-101 sensitivity was 92.5%, the specificity was 97.5%, the PPV was 97.3%, the NPV was 92.8% and the diagnostic accuracy was 95%. The ROC curve (AUC) for miR-101 was 0.9(95% CI: 0.909 — 1.002), indicating perfect validity discrimination of the HCC population from both cirrhotic patients and healthy individuals (Table 6; Fig. 5).

**Table 6:** Potential diagnostic of serum AFP and miR-101 as markers for HCC using ROC curve analysis.

Variable	Cut Off (ng/ml)	AUC (95%CI)	Sensitivity (%)	Specificity (%)	P.P.V (%)	N.P.V (%)	D.A (%)
AFP	7.0	0.6478 **0.524 — 0.770**	60%	67%	64%	62%	63.7%
miR-101 (2^-ΔC^T)	**1.0 FC**	0.9556 **0.909 — 1.002**	92.5%	97.5%	97.3%	92.8%	95%

*PVP* positive predictive value, *PVN* negative predictive value, *DA* diagnostic accuracy, *AUC* the area under the curve, *CI* confidence interval, *FC* Fold change.

### Pearson correlation of serum miR-101 and AFP levels with clinicopathological features of HCV‑induced HCC groups

The investigation of the Person’s correlation coefficient between miR-101, AFP, and other clinicopathological markers was performed. Regarding AFP, our findings do not show any significant correlations with the listed clinical parameters (ALT AST, Albumin, T. Bilirubin, D. Bilirubin, T. Protein, Focal Lesion number and size, BCLC, Child–Pugh Score). On the other hand, miR-101 expression levels indicated that there was a statistically significant strong positive correlation with Focal Tumor characteristics including lesion number (r = 0.411, *P* = 0.0080), lesion size (r = 0.451, *P* = 0.0030), BCLC (r = 0.5600, *P* = 0.0002), Child–Pugh Score (r = 0.5440, *P* = 0.0002). However, no statistically significant correlation was found among the studied miRNAs and liver enzymes and functions in the HCC group ((*P* > 0.05) (Table 7).

**Table 7:** Person’s correlation coefficient analysis of studied AFP and miR-101 in relation to biochemical parameters in the HCV-associated HCC investigated group.

Variable	AFP		miR-101	
	*r*	*P*. Value	*r*	*P*. Value
ALT	− 0.1090	0.5050	− 0.2760	0.0850
AST	− 0.0890	0.5850	− 0.2770	0.0830
Albumin	0.1250	0.4410	0.2670	0.0950
T. Bilirubin	0.2030	0.2090	0.0740	0.6490
D. Bilirubin	0.0280	0.8630	− 0.0450	0.7850
T. Protein	0.2330	0.1480	0.1710	0.2910
Focal Lesion No	− 0.1750	0.2810	0.4110	**0.0080**
Focal Lesion Size	− 0.1870	0.2480	0.4510	**0.0030**
BCLC	− 0.1340	0.4090	0.5600	**0.0002**
Child–Pugh Score	0.0080	0.9630	0.5440	**0.0002**

P = P-value, *r* = Correlation Coefficient, r = -1 Perfect negative correlation, r < 0 no correlation, r = 0: < 0.5 Weak positive correlation, r = 0.5 fair correlation, r =  > 0.5: < 0.75 Moderate positive correlation, r = 0.75 strong correlation, r = 1 Perfect positive correlation; For the statistical variances between the groups under examination, bold values are emphasized.

## Discussion

The poor prognosis of HCC is primarily attributed to the lack of suitable biomarkers for early diagnosis, along with limited treatment options such as liver resection, transplantation, or other therapies for unresectable liver cancer, contributing to low HCC-associated survival rates^28^. Hence, there is an urgent need to discover reliable non-invasive biomarkers with specific early predictive values for detecting HCC. miRNAs, a special class of non-coding RNA, have shown encouraging results in the early identification of HCV-HCC. Accordingly, the objective of this study was to investigate the diagnostic potential of miR-101 for HCV-HCC as an alternative biomarker for AFP by estimating fold-change expression levels among HCC and cirrhotic groups compared with control groups, ultimately improving diagnostic methods for the early detection and treatment of HCV-HCC.

Considering the enrollment strategy conducted in our study, 100 subjects were enrolled and classified into three main groups: HCV-HCC, HCV liver cirrhosis, and HCV-free healthy control volunteers. Male patients predominated in the HCC, cirrhosis, and healthy control groups. The prevalence of HCC in men could be attributed to the contribution of several factors, including testosterone, dihydrotestosterone, androgen, and proteomic deficiencies, in pathogenesis and therapy^29^. The mean age of healthy controls was, on average, younger than that of patients with HCC and cirrhosis, in contrast to both HCV-related HCC and HCV-related liver cirrhosis, which is quite similar (~ 60.90 ± 6.10), indicating that individuals in this age group are more susceptible to developing liver cirrhosis and subsequently HCC (Table 1). This aligns with the well-established understanding that HCC incidence increases with age, particularly in older individuals^30^.

For biochemical profile analysis (Table 3), ANOVA indicated a non-significant difference in ALT and AST levels between the tested groups (*P* = 0.2055 and 0.0612, respectively). These findings agree with those of a study that reported no statistically significant difference in ALT and AST levels between the control and infected groups (*P* > 0.001)^31^. Furthermore, in terms of serum TB and DB levels, patients with HCC and cirrhosis had significantly higher levels (*P* < 0.0001). In comparison, ALB levels were significantly lower (*P* < 0.0001) in the experimental group than in the control group, which is consistent with^32^. The abnormal serum biochemical parameters observed in patients with HCC and cirrhosis may be linked to the presence of a liver tumor, which can result in significant damage to surrounding hepatocytes^33^. This damage is caused by the physical disruption of the liver tissue and interference with normal hepatocyte function. Additionally, the tumor promotes the development of new blood vessel networks (angiogenesis), often leading to areas of low oxygen levels (hypoxia)^34^ within the tumor and surrounding liver tissue, further exacerbating damage. These effects are compounded by conditions such as cirrhosis and chronic active hepatitis^35^, which involve continual hepatocyte necrosis or cell death. The ongoing cycle of hepatocyte damage and death contributes to disease progression.

*Regarding HCC cases and their stages at diagnosis,* as presented in Table 4, our research findings indicate that 42% of HCC patients were diagnosed at stage A (*early stage*), which is defined by the presence of a single tumor of any size or up to 3 tumors less than 3 cm in size. with a performance status of 0 (PS 0) and were active with normal liver function (Child–Pugh A or B). Additionally, our study showed that 22% of patients were diagnosed at stage B (*intermediate stage*), characterized by multiple tumors in the liver, but still having a good performance status of 0 (PS 0) and normal liver function(Child–Pugh A or B), and 35% were at stage C (*advanced stage*), marked by symptomatic tumors and invasive and/or metastatic disease with compromised performance status (PS 1 or 2), but still maintained relatively good liver function according to (Child–Pugh A or B), to the BCLC staging system^24^. Our findings align with those of a multicenter study conducted in Spain^24^, where 49.8% of 705 cases of HCC occurred in stage A, 19.8% in stage B, 18.8% in stage C, and 11.6% in stage D. The limited treatment options for these stages underscore the significance of early detection in enhancing therapeutic response and significantly improving survival rates^36^.

The extensively employed *AFP serum biomarker* levels in the diagnosis and monitoring of HCV-related HCC are well-established^37,38^, In the present study, ANOVA analysis revealed statistically significant elevated AFP levels in HCC compared to both the cirrhotic and control groups (Table 3; Fig. 1). The ANOVA analysis and the unpaired t-test comparison demonstrated distinct statistically significant differences in mean serum AFP levels among patients with HCC and those with cirrhosis, compared to the control group (*P* = 0.0259), with the highest level of AFP reported in the HCC group (88.99 ± 186.9). Our findings are consistent with those of Taura et al*.*, who reported that in HCV patients with cirrhosis, AFP level ≥ 6 ng/mL was associated with the development of HCC in multivariate analysis^39^.

The effectiveness of serum AFP as a monitoring test has been a topic of ongoing debate^40^. In the 2010 guidelines published by the American Association for the Study of Liver Diseases, AFP was not recommended for use in identifying HCC^41^. Similarly, in their 2012 recommendations, the European Association for the Study of the Liver stated that AFP is not sufficiently sensitive or specific for use as a diagnostic instrument^42^. Regarding the *diagnostic performance of AFP* as a discriminating marker for HCC versus cirrhotic populations, ROC curve data analysis showed low validity AUC = 0.6 (95% CI, 0.524 — 0.770) with 60% sensitivity, 67% specificity, a positive predictive value of 64%, and a negative predictive value of 62% at a cutoff value of 7 ng/dl (Table 6; Fig. 4). Our results are consistent with those of a study that reported that the sensitivity of AFP for identifying HCC ranged from 41 to 65%^43,44^. The suboptimal diagnostic performance of AFP in early stage HCC detection could be attributed to nonspecific increased levels in different scenarios, such as chronic hepatitis B infection, fatty liver disease, increased serum AST/ALT ratio, elevated serum ferritin, Mallory bodies in liver biopsies, liver disease with viral etiology, low albumin level, larger tumor size, multinodular HCC, and the presence of vascular invasion, which are also associated with elevated AFP levels in patients with HCC^45–47^. Subsequently, AFP alone may not be sufficient for the diagnosis of HCC and should be interpreted in the context of other clinical and imaging findings as well as other biomarkers. Because of their integral role in regulating pathways linked to cancer, miRNAs have attracted attention for their clinical relevance as pathological markers for the early diagnosis, classification, and prognostic stratification of HCC patients^27^.

Among these, miR-101 is involved in cancer-related biological processes, including proliferation, apoptosis, angiogenesis, drug resistance, invasion, and metastasis. The great circulation stability of miRNAs makes them ideal noninvasive biomarkers, especially for the diagnosis of pre-symptomatic disorders and initial stages, opening the way for new diagnostic and therapeutic approaches^46^. In this study, *we assessed the expression pattern of circulating miR-101 in HCC*. RT-qPCR—FC assessment of miR-101 expression profiles in serum across HCV-HCC and HCV-liver cirrhosis compared to the control revealed a statistically significant difference (*P* < 0.0001). Based on the average FC values, serum miR-101 levels in HCC patients reported a highly statistically significant upregulated expression (*P* < 0.0001) of miR-101 when compared with the controls (2.427 ± 0.597 vs. 1.179 ± 0.13, representing a 2.4 -fold increase). In contrast, cirrhotic patients reported a non-significant downregulation of miR-101 expression compared to controls (1.062 ± 0.264 vs. 1.179 ± 0.13). Furthermore, according to the unpaired t-test, comparing the mean serum values of miR-101 between the HCC and cirrhotic groups revealed a significant difference in miR-101 levels (*P* < 0.0001) (Table 5; Fig. 3). These findings suggest that dysregulation of miR-101 expression during HCV-related HCC is associated with the onset and progression of HCC, as they can function as tumor suppressor genes, oncogenes, or regulatory molecules^48^. Moreover, the increased miR-101 levels in HCC compared to its downregulation in cirrhosis may be attributed to the release of miR-101 into circulation due to tissue damage. This is similar to the cases of miR-122 and miR-192, which exhibit evidence of liver degeneration in mice following medication therapy^49^. Therefore, profiling the expression levels of miR-101 could be used to discriminate HCC from cirrhotic and control groups. This may offer a valuable noninvasive biomarker for identifying HCV-induced HCC. In contrast to our findings, multiple independent studies have demonstrated that miR-101 and are downregulated in a context-dependent manner including viral-induced HCC (particularly in HBV-related cases^50^, and multiple malignances^51–56^. The discrepancy in miR-101 expression (upregulation in our research versus downregulation in previously mentioned studies) could be due to several factors including differential expression in HCV vs. HBV contexts, as the distinct biological mechanisms and pathways activated by these hepatotropic viruses may influence miRNA expression differently. A meta-analysis of studies on miR-101 expression in HCC, focusing on the differences between HBV and HCV infection, is needed to clarify the discrepancies in findings. Also, miR-101 can target different genes in a context-dependent manner. For instance, in gastric cancer, miR-101 targets the SOCS2 oncogene^55^, leading to growth suppression. Similarly, in lung cancer, miR-101 targets DNMT3a^52^, resulting in DNA hypomethylation. The regulatory role of miRNAs over numerous targets involved in signal transduction pathways contributes to the intricate nature of their involvement in hepatocarcinogenesis^57^. In HCV-induced HCC, miR-101 may target a gene that leads to its upregulation. Further investigation of the specific targets of miR-101 in the HCV-induced HCC model would be helpful. Additionally, sample type (serum vs. tissue), where the expression levels of MiR-101 may exhibit different expression profiles in serum compared to tissue samples^58^. The target population for our study was Egyptian patients, which may have unique genetic or environmental or lifestyle factors that influence miR-101 expression compared to other regions. It is important to consider the genetic and environmental factors specific to the Egyptian population that may contribute to the observed upregulation of miR-101.

*The diagnostic performance of circulating miR-101 biomarkers for discriminating HCC* was also estimated using ROC curve and CI analysis. Our results showed that miR-101 sensitivity as a single marker for HCV-induced HCC of 92.5%, compared to AFP, which exhibited a sensitivity of only 60%, a specificity degree of 97.5%, a positive predictive value was 97.3%, the negative predictive value of 92.8%, and the diagnostic accuracy of the test of 95%. The AUC of the ROC curve analysis was 0.95; 95% CI: 0.524—0.770) for miR-101 in contrast to the AUC of AFP which was 0.6478; 95% CI: 0.909 -1.002 (Table 6; Fig. 5). These findings suggest that serum miR-101 has the potential to be a sensitive and promising biomarker for the early diagnosis of HCC in Egyptian patients with HCV when compared to AFP levels. While the current analysis evaluates the individual performance of miRNA-101 as a standalone proposed diagnostic tool for HCV-induced HCC, this approach offers several advantages in clinical applications, such as cost-effectiveness for patients and streamlined implementation. However, the combined utilization of miRNA-101 and AFP biomarkers may potentially enhance the overall diagnostic efficacy. Further research is necessary to investigate this possibility.

In addition, *the correlations between AFP, miR-101, and other biochemical markers of HCV-related HCC* were evaluated. The findings of the Person correlation coefficient indicated that there was no statistically significant association between serum AFP and other biochemical parameters (ALT, AST, Albumin, T. Bilirubin, D. Bilirubin, T. Protein) in the HCC group (Table 7). This lack of correlation can be crucial to consider when utilizing AFP as a diagnostic tool for HCC because it indicates that AFP levels in the blood cannot adequately represent the degree of liver damage or inflammation in a patient with HCC caused by HCV (evidenced by a 60% sensitivity rate). Therefore, to thoroughly assess liver function and diagnose HCC, other diagnostic methods (such as miR-101) and biochemical markers may need to be used in addition to AFP.

In our study on HCC, we explored the miR-101 association between focal tumor characteristics. Our results demonstrated several significant correlations: higher miR-101 expression levels were associated with increased multiple tumor lesions (r = 0.411, *P* = 0.0080), larger tumor size (r = 0.451, *P* = 0.0030), and more advanced disease stages based on the BCLC staging system (r = 0.5600, *P* = 0.0002) and Child–Pugh score (r = 0.5440, *P* = 0.0002). Consistent with other studies, Xuecheng found that the high expression of miR-101 was linked to larger tumor size and advanced pathological stage, and that there is a potential correlation between miR-101 expression levels and the Child–Pugh score^59^. Elevated levels of miR-101 in HCC are associated with a higher number and larger size of lesions, as well as advanced BCLC staging and Child–Pugh score. This association suggests that monitoring miR-101 levels could help to predict the presence of multiple tumor lesions, estimating lesion size non-invasively, improving the accuracy of BCLC staging, and assessing liver disease severity more effectively in combination with routine tests. Although our cross-sectional study has demonstrated a significant correlation between miR-101 expression levels and various tumor characteristics at a single time point, suggesting that miR-101 may be indicative of the tumor’s aggressiveness and overall disease severity. However, the inherent limitations of a cross-sectional design restrict our ability to evaluate the prognostic value of miR-101. Unlike cohort or longitudinal studies, cross-sectional analyses do not allow for follow-up assessments, which are essential for determining how miR-101 levels influence long-term outcomes or survival rates. Cohort studies with longitudinal monitoring and increased participant numbers are necessary to further examine the potential of miR-101 as a prognostic biomarker. These investigations should evaluate the relationship between miR-101 expression levels and treatment outcomes, as well as the broader applicability of findings related to miR-101. Additionally, exploring the biological mechanisms of miR-101 in tumor biology can clarify its potential as a prognostic and therapeutic target. While we report a significant correlation between the expression levels of miR-101 and various tumor parameters, combining the analysis of miR-101 with other well-established biomarkers may enhance prognostic precision and offer a more comprehensive understanding of patient prognosis. As a result, integrating miR-101 measurement into clinical trials could provide valuable insights into its efficacy as a prognostic indicator and its potential for informing treatment strategies.

While miRNAs regulate gene expression by binding to target mRNAs, their stability is crucial for their functional role^60^. Extracellular vehicles (EVs) are nano-sized, lipid bilayer-enclosed structures that are naturally secreted by cells into the extracellular space^61^, as tiny diameter, excellent biocompatibility, low immunogenicity, high circulation stability, intrinsic targeting capacity^62^. These vesicles are recognized as bioactive carriers that mediate a variety of biological processes, facilitating intercellular communication by transporting diverse cellular components, including miRNAs^61^. EVs protect miRNAs from extracellular enzymatic degradation through various mechanisms as encapsulation within their lipid bilayer membrane, association with RNA-binding proteins, or lipoproteins, inside EVs, thereby enhancing miRNA stability and facilitating their selective packaging into EVs^60^.

Cancer cells, particularly HCC, release a greater quantity of EVs compared to healthy cells. These EVs carry a unique molecular signature reflecting the physiological state of their parent cells, including HCC cells^63^. contributing to cancer progression and metastasis^61,64^. Consequently, EVs and their integrated molecular profiles hold significant potential as stable biomarkers for HCC diagnosis, particularly in the context of miR-101.

It was reported that HCC-derived EVs demonstrate a differential expression of specific miRNAs compared to healthy cells, that serve as promising biomarkers for HCC diagnosis and prognosis^61^. So, analyzing miR-101 levels in EVs from bodily fluids could be a valuable diagnostic tool. As, reduced miR-101 in EVs from HCC patients’ blood, urine, or saliva may indicate early diagnosis, development, and disease progression. This approach is consistent with the concept of liquid biopsy, which relies on the ready availability of EVs `in diverse bodily fluids^63^, thereby enables non-invasive sampling techniques that can offer insights into the dynamics of HCC and patient responses to therapy^65^. Further research should investigate the potential of miR-101 as an EV-based biomarker for accurate, stable biomarkers for early diagnosis and HCC monitoring. This includes profiling miR-101 in HCC cells and HCC-derived EVs from patients versus controls, determining the correlation between miR-101 expression in EVs and HCC disease status and outcomes, exploring the mechanisms of miR-101 packaging into EVs, and combining miR-101 with other EV-associated biomarkers (as miR-483-5p or miR-638) may also improve HCC diagnosis^64^.

*The discrepancies* between the outcomes of other studies and our investigation might be attributed to either varied miRNA expression based on the study design, etiology, stage, type of therapy, sources, and techniques of sample collection, usage of platforms with varying degrees of sensitivity, regional diversity in HCC among different populations, and Other than genotype 4, additional risk factors for HCC besides HCV (alcohol-associated liver disease, non-alcoholic fatty liver disease, aflatoxin exposure, smoking, and inherited liver diseases). Although our study provides valuable insights into the potential of miR-101 as a biomarker for HCC diagnosis in Egyptian patients, the comparatively small sample size of 80 patients restricts the generalizability of our findings. Therefore, future research using a larger and more diverse sample population is essential to validate and extend our results. This study may serve as a basis for future research.

## Conclusion

In this study, we examined the serum expression patterns of miR-101 in Egyptian patients with HCV-associated HCC compared to those in HCV-liver cirrhosis patients and healthy controls. According to our investigation, altered levels of miR-101 expression were observed in patients with Chronic HCV infection, showing upregulation in HCC and downregulation in liver cirrhosis, highlighting its role in the disease progression and the potential use of miR-101 expression as a biomarker for HCC monitoring. Additionally, the present study clearly shows that miR-101 can be utilized as a non-invasive biomarker for early detection of HCV-associated HCC progression either standalone or with other miRNAs or AFP to discriminate HCV-associated HCC progression, particularly in the early stages, in comparison to other HCV-related liver cirrhosis together with the control group, with a higher sensitivity (92.5%) and specificity (97.5%) than AFP. Additionally, miR-101 expression levels showed a positive correlation with lesion number, size, BCLC staging, and Child–Pugh score in HCC, highlighting the potential of miR-101 levels to predict multiple tumor lesions, estimate lesion size non-invasively, and enhance BCLC staging accuracy. We believe that this study is the first in Egypt to correlate the serum expression profiles of miR-101 with HCV-associated liver HCC and liver cirrhosis. Further investigations are required to confirm the potential of miR-101 as a promising diagnostic and prognostic biomarker for HCC in the clinical setting. Additionally, further studies should be conducted to better understand the molecular mechanisms underlying the function of miR-101 in HCC and the biology of liver cirrhosis. Finally, to completely comprehend the biological importance of miR-101 in the development of HCC in a range of individuals with different HCV genotypes and other risk factors other than HCV for HCC, further studies should be conducted.

## Data Availability

The datasets created or analyzed during this work are involved in this manuscript and will be available from the corresponding author on reasonable request.

## Abbreviations

miRNA: MicroRNA
AFP: α-Fetoprotein
HCV: Hepatitis C virus
HCC: Hepatocellular carcinoma
LC: Liver cirrhosis
HC: Healthy control
CHCV: Chronic hepatitis C virus
CT: Computed tomography
BCLC: Barcelona Clinic Liver Cancer
RT-qPCR: Quantitative reverse transcription PCR
RT: Reverse Transcription
ROC: Receiver operator characteristic curve
AUC: Area under the curve
PVP: Positive predictive value
PVN: Negative predictive value
DA: Diagnostic accuracy
CI: Confidence interval
FC: Fold change
EASL: European association for the study of the liver
AASLD: American association for the study of liver diseases
